# Combining Biomimetic Block Copolymer Worms with an Ice‐Inhibiting Polymer for the Solvent‐Free Cryopreservation of Red Blood Cells

**DOI:** 10.1002/anie.201511454

**Published:** 2016-01-28

**Authors:** Daniel E. Mitchell, Joseph R. Lovett, Steven P. Armes, Matthew I. Gibson

**Affiliations:** ^1^ Department of ChemistryUniversity of WarwickGibbet Hill RoadCoventryCV4 7AlUK; ^2^ Department of ChemistryUniversity of SheffieldSheffieldS3 7HFUK

**Keywords:** biomaterials, block copolymers, cryopreservation, micelles, polymers

## Abstract

The first fully synthetic polymer‐based approach for red‐blood‐cell cryopreservation without the need for any (toxic) organic solvents is reported. Highly hydroxylated block copolymer worms are shown to be a suitable replacement for hydroxyethyl starch as a extracellular matrix for red blood cells. When used alone, the worms are not a particularly effective preservative. However, when combined with poly(vinyl alcohol), a known ice‐recrystallization inhibitor, a remarkable additive cryopreservative effect is observed that matches the performance of hydroxyethyl starch. Moreover, these block copolymer worms enable post‐thaw gelation by simply warming to 20 °C. This approach offers a new solution for both the storage and transport of red blood cells and also a convenient matrix for subsequent 3D cell cultures.

Donor cells and tissue are essential components of modern medicine. For example, 30 million units of blood are annually transfused in the USA, and up to 100 pints (57 liters) of blood are required for a single trauma victim.^1^ Leukemia treatment requires donor bone marrow, and emerging regenerative medicines (e.g., stem‐cell treatments) require a constant supply of cells and the logistical infrastructure to transport them.^2^ However, this is complicated by the finite lifetime of isolated cells.^3^ Red blood cells can be kept for a maximum of 42 days (but typically for shorter periods), platelets for 8 days, and donor organs for just a matter of hours. In principle, cryopreservation (freezing to reduce the rate of cellular degeneration) can be used to enable the storage and transport of cells and tissue.^4^ Current state‐of‐the‐art strategies for cryopreservation require the addition of large amounts of water‐miscible organic solvents, such as glycerol or DMSO, to promote vitrification (ice‐free state) or dehydration.^5^ There are several problems with this approach, not least solvent toxicity, and the need (and challenges) of removing all traces of such solvents before transfusion. There are also many cell types (for medicine and basic biosciences) that are challenging to store using current methods.^6^

A major cause of damage in cellular cryopreservation is attributed to ice recrystallization (growth) during thawing. Ice‐recrystallization inhibitors, such as antifreeze (glyco)proteins (AF(G)Ps), enhance cellular cryopreservation but are challenging to synthesize and have biocompatibility issues.^7^ Gibson and co‐workers have previously described the use of synthetic polymers as mimics of AF(G)Ps to enhance cell recovery after thawing.^8^ However, in both cases (polymer and AF(G)P), it was necessary to add a supplementary extracellular cryoprotectant. Typically, hydroxyethyl starch (HES) is used as a non‐toxic alternative to solvents,^9^ but this biopolymer does not come as a pure product: it has variable degrees of hydroxyethyl moieties and a broad molecular‐weight distribution. Furthermore, HES has recently been partially withdrawn from clinical use owing to a possible increase in mortality for critically ill patients.^10^ To the best of our knowledge, no synthetic mimics of HES have been evaluated for cryopreservation. Conversely, the use of synthetic copolymer gels as mimics of the extracellular matrix for 3D cell cultures is a rapidly developing field. Armes and co‐workers have demonstrated that block copolymer worms are potentially useful matrices for cell culture studies as they can be readily switched between fluid and gel phases by a change in temperature, enabling facile sterilization by cold ultrafiltration.^11^

The aim of this study was to investigate the use of diblock copolymer worms as wholly synthetic biomimetic alternatives to HES for cellular cryopreservation and to examine their additive effects when used in combination with polymeric ice‐recrystallization inhibitors. The feasibility of thermally triggered hydrogelation after thawing, which is highly desirable for tissue‐engineering applications, was also explored.

The first step was to prepare the poly(glycerol monomethacrylate)‐*block*‐poly(2‐hydroxypropyl methacrylate) (PGMA‐PHPMA) block copolymer worms by polymerization‐induced self‐assembly (PISA; Figure 1 A).11b More specifically, RAFT aqueous dispersion polymerization of HPMA was conducted using a PGMA_56_ macro‐CTA (CTA=chain transfer agent) to target PGMA_56_‐PHPMA_155_ worms, as previously reported by Armes and co‐workers (see the Supporting Information for further experimental and characterization details).11a, 12 These worm gels exhibited thermoresponsive behavior, undergoing degelation upon cooling to 5–10 °C by a reversible worm‐to‐sphere transition.11a This order–order morphological transformation may provide a useful trigger for the construction of 3D cell‐seeded gels (see below). Oscillatory rheology studies of a 10 % w/w aqueous worm gel indicated a critical gelation temperature (CGT) of approximately 12 °C, as judged by the intersection of the *G*′ and *G*′′ curves (see the Supporting Information, Figure S3). A representative TEM image obtained upon drying a dilute aqueous dispersion of PGMA_56_‐PHPMA_155_ worms at 20 °C is shown in Figure 1 B.

**Figure 1:**
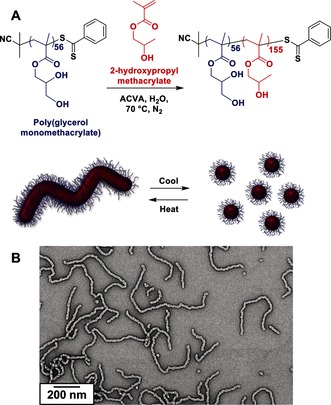
A) Synthetic route for the RAFT aqueous dispersion polymerization of HPMA using a water‐soluble PGMA_56_ macro‐CTA to form PGMA_56_‐PHPMA_155_ diblock copolymer worms. Such worms form a soft, free‐standing aqueous hydrogel at 20 °C, but undergo a reversible worm‐to‐sphere transition upon cooling below 12 °C. B) Representative TEM image of the PGMA_56_‐PHPMA_155_ diblock copolymer worms after drying a dilute aqueous dispersion at 20 °C.

Ice recrystallization inhibition (IRI) is a unique (and rare) property exhibited by certain macromolecules.^13^ To evaluate whether the PGMA_56_‐PHPMA_155_ worms exhibited IRI behavior, they were assayed using the standard “splat” test and compared to poly(vinyl alcohol) (PVA), which is a potent IRI‐active polymer.^14^ Briefly, this assay involves creating a 10 μm thick wafer of small ice crystals, which are then annealed at −6 °C for 30 min before determining the mean largest grain size (MLGS) of the ice crystals (with smaller ice crystals indicating higher activity). The results of this assay are shown in Figure 2 A. The PGMA_56_‐PHPMA_155_ worms showed no activity even at 20 mg mL^−1^, which is comparable to the negative poly(ethylene glycol) (PEG) control and also HES (see below). In contrast, PVA is highly active even at 1.0 mg mL^−1^, which is consistent with our earlier studies.8a Differential scanning calorimetry studies confirmed that an aqueous dispersion of PGMA_56_‐PHPMA_155_ worms does indeed crystallize when cooled (as indicated by the strong exotherm at ca. −20 °C; in addition, a melting temperature of around −5 °C was also observed). This observation is important because many current cryopreservation solutions rely on vitrification by the addition of large quantities of (toxic) organic solvents. If vitrification had occurred, a much weaker (or zero) exotherm would have been observed upon cooling as a result of the formation of a glassy, rather than a crystalline state.

**Figure 2:**
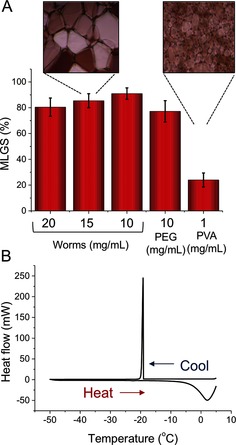
Effect of the worms and PVA on ice formation/growth. A) IRI activity of the worms and PVA. Data reported relative to the PBS control. Inset images: 500 μm. B) Differential scanning calorimetry of worm dispersions (20 mg mL^−1^) with cooling at 10 °C min^−1^ and thawing at 2 °C min^−1^.

The above data clearly show that the PGMA_56_‐PHPMA_155_ worms cause neither ice growth nor nucleation. This makes them a good candidate to act as wholly synthetic non‐penetrative cryoprotectants like certain biopolymers, such as hydroxyethyl starch, which can form a hydrated matrix around cells. Red blood cells were chosen for cryopreservation studies, as there is an urgent need to improve their long‐term storage without recourse to toxic organic solvents. Our previous studies had demonstrated that the addition of IRI‐active PVA increases cell recovery by minimizing ice‐induced damage.8a Red‐blood‐cell recovery can be determined by comparing the relative degree of hemolysis to a positive control—this serves as a key clinical indicator of their post‐thaw utility. Preliminary screening studies indicated that the PGMA_56_‐PHPMA_155_ worms were non‐hemolytic towards red blood cells at concentrations up to 20 mg mL^−1^. Accordingly, a 5 wt % aqueous dispersion of PGMA_56_‐PHPMA_155_ worms was added to the red blood cells (5×10^6^ cells mL^−1^) in the presence and absence of 1 mg mL^−1^ PVA (this optimal PVA concentration inhibits ice growth without inducing dynamic ice shaping).8a These aqueous mixtures were rapidly frozen by immersion in liquid nitrogen and stored above liquid N_2_ for three days, followed by slow thawing at 4 °C. This thawing protocol was chosen to maximize cell stress, thus providing a stringent test of the cryopreservative performance of this new PVA/PGMA_56_‐PHPMA_155_ worm formulation. It is also representative of the environment typically used for large‐volume cell freezing for which temperature gradients are known. Moreover, this thawing temperature ensures that no worm gel formation occurs, as it is below the CGT of 12 °C. The results of these freeze–thaw experiments are shown in Figure 3 relative to PBS and PVA‐only controls.

**Figure 3:**
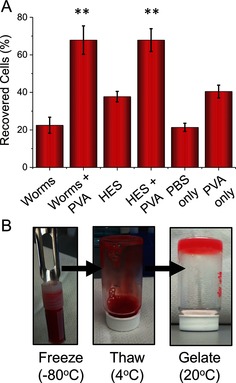
Cryopreservation of red blood cells. A) Cell recovery (1/hemolysis) after storage above N_2 (liq)_ for three days. [Worms]=5 wt %, [HES]=20 wt %, [PVA]=1 mg mL^−1^. Error bars represent the standard deviations from a minimum of three repeats. B) Gelation capability following a freeze/thaw cycle. After thawing at 4 °C, the blood solutions formed free‐standing cells when heated to 20 °C.

The PGMA_56_‐PHPMA_155_ worms alone resulted in just 20 % cell survival after thawing, which is somewhat lower than the optimized 40 % cell survival achieved in the presence of HES and not statistically different from that of PBS alone. The addition of PVA to HES produced a substantial increase in cell recovery of up to 70 %, which is consistent with the hypothesis that inhibiting ice growth is key to effective cryopreservation. PVA alone only enabled 40 % cell recovery, highlighting the importance of a secondary hydrated component. Remarkably, the addition of PVA to the PGMA_56_‐PHPMA_155_ worms gave 68 % recovery, which is statistically indistinguishable to that of the HES/PVA system. There was no evidence for any hemagglutination or abnormal cell morphologies. This is the first demonstration that a wholly synthetic (polymer or otherwise) formulation can be used to achieve efficient cell cryopreservation. Clearly, there is huge scope for further optimization as well as the incorporation of additional functionality, such as cell‐adhesion motifs or fluorescent labels, by rational design. Moreover, the potential to achieve in situ aqueous gelation immediately after thawing is highly desirable for tissue‐engineering applications (if not for blood itself). For example, Figure 3 B shows digital photographs of a whole blood sample that had been cryopreserved, thawed, and then heated above the CGT of the worms, demonstrating the rapid formation of a gel rich in red blood cells directly from the cryopreservation mixture described herein.

In summary, we have demonstrated that diblock copolymer worm gels are the first synthetic alternative to biopolymers (such as hydroxyethyl starch) for the solvent‐free cryopreservation of red blood cells, with particular efficacy being achieved when combined with an ice‐recrystallization inhibitor such as poly(vinyl alcohol). After initial thawing at 4 °C, the copolymer worms retained their ability to form free‐standing gels upon warming to room temperature, suggesting an attractive one‐pot solution for future whole blood cryopreservation and tissue‐engineering applications.

## Supporting information

As a service to our authors and readers, this journal provides supporting information supplied by the authors. Such materials are peer reviewed and may be re‐organized for online delivery, but are not copy‐edited or typeset. Technical support issues arising from supporting information (other than missing files) should be addressed to the authors.

Supplementary
