# Supplementary material for: Combining Biomimetic Block Copolymer Worms with an Ice‐Inhibiting Polymer for the Solvent‐Free Cryopreservation of Red Blood Cells
Source: Angew Chem Int Ed Engl. 2016 Jan 28;55(8):2801–4. doi: 10.1002/anie.201511454 (PMC4770432; doi:10.1002/anie.201511454)
Supplement: Supplementary file 1 — Supplementary [file ANIE-55-2801-s001.pdf]

Angewandte Chemie
Eine Zeitschrift der Gesellschaft Deutscher Chemiker

Supporting Information

Combining Biomimetic Block Copolymer Worms with an Ice-Inhibiting Polymer for the Solvent-Free Cryopreservation of Red Blood Cells

*Daniel E. Mitchell, Joseph R. Lovett, Steven P. Armes, and Matthew I. Gibson**

anie_201511454_sm_miscellaneous_information.pdf

Additional Copolymer Characterization Data

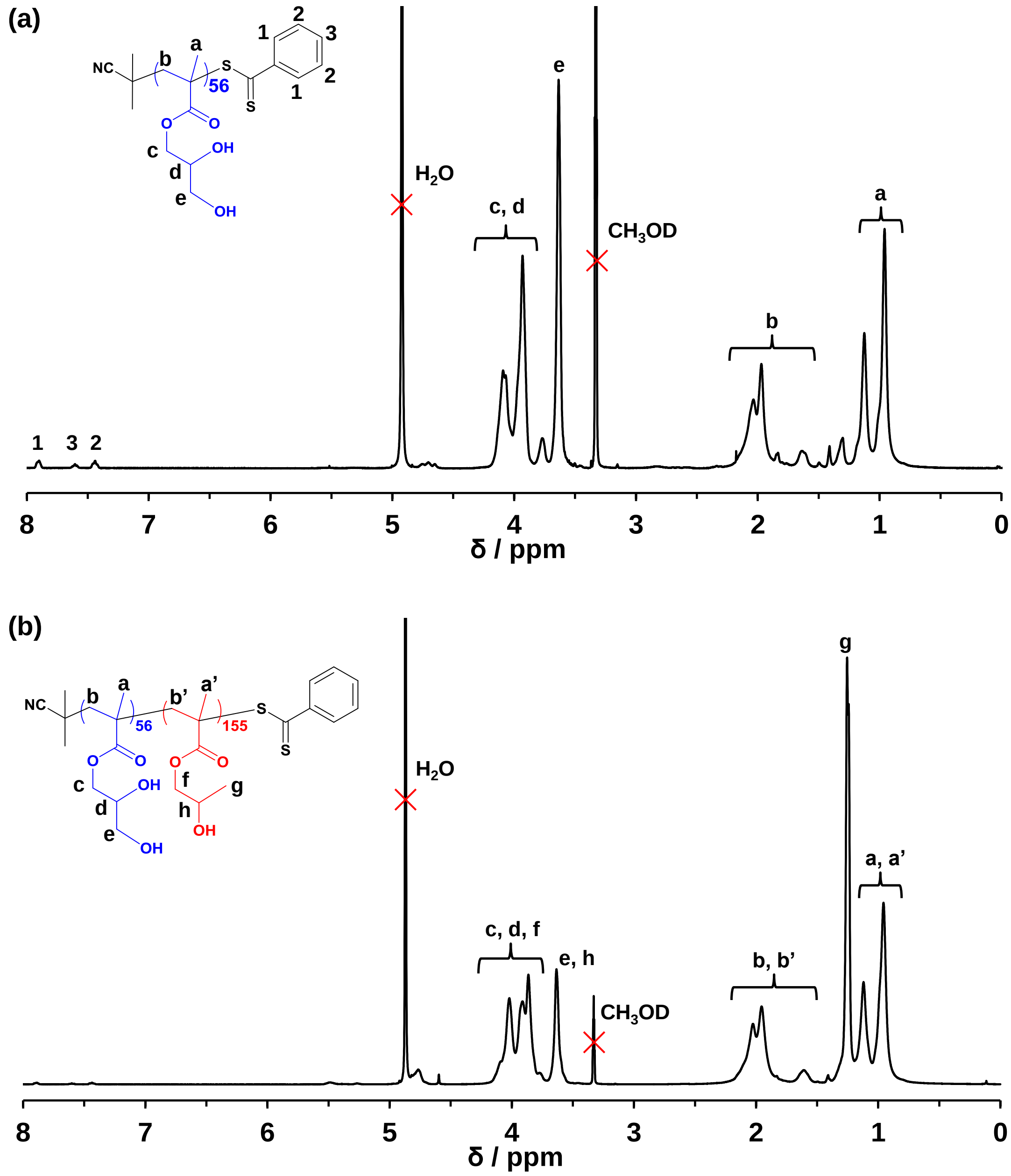

Figure S1. Assigned ^{1}H NMR spectra recorded for (a) freeze-dried $PGMA_{56}$ macro-CTA redissolved in CD_3OD and (b) freeze-dried $PGMA_{56}$-$PHPMA_{155}$ diblock copolymer redissolved in CD_3OD.

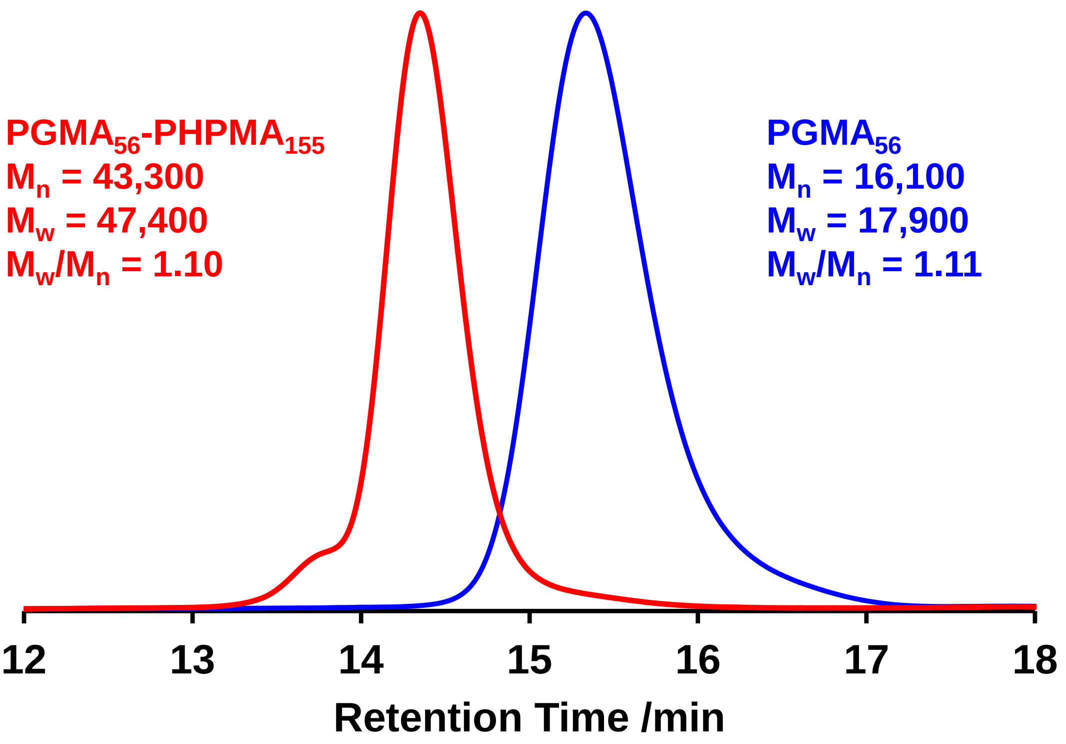

Figure S2. DMF GPC traces obtained for the $PGMA_{56}$ macro-CTA (blue curve) and $PGMA_{56}$-$PHPMA_{155}$ diblock copolymer (red curve) against a series of near-monodisperse PMMA calibration standards.

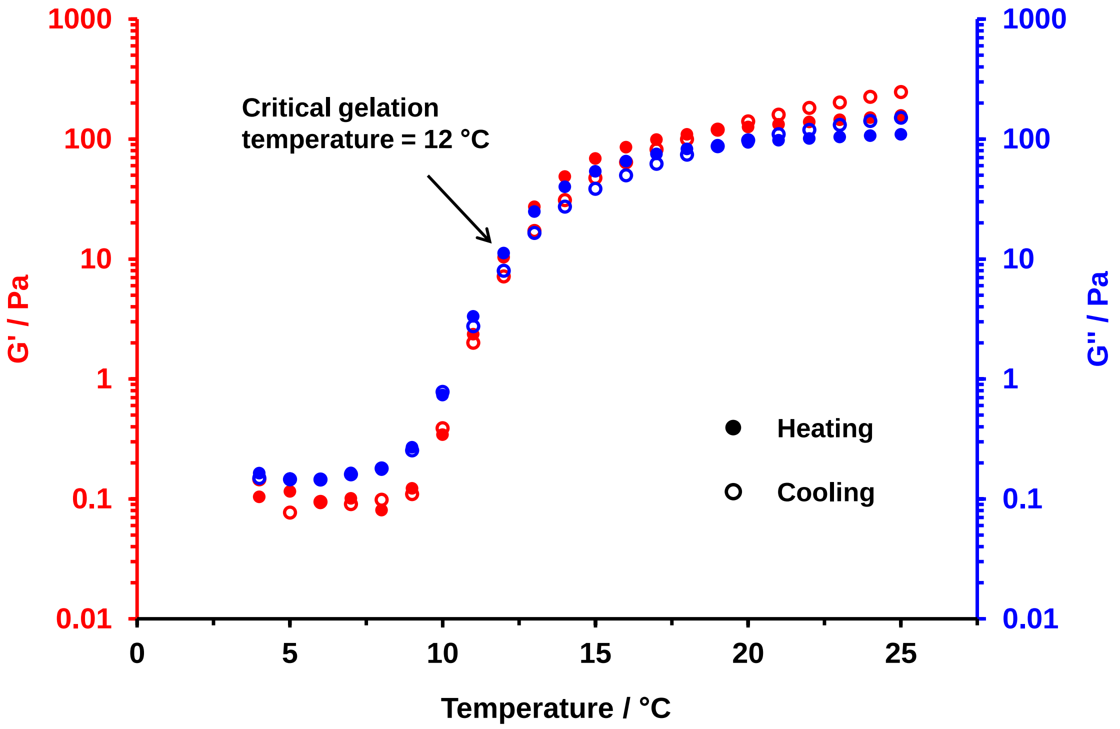

Figure S3. Temperature dependence of the storage modulus (G'; red data sets) and loss modulus (G''; blue data sets) for a 10 % w/w aqueous dispersion of $PGMA_{56}$-$PHPMA_{155}$ diblock copolymer nano-objects. Conditions: frequency = 1.0 rad s^{-1}; applied strain = 1.0 %. The cross-over of the G' and G'' curves indicates a critical gelation temperature of around 12 °C.

Experimental Section

Physical and Analytical Methods

NMR spectroscopy. ^{1}H NMR spectra were recorded using a 400 MHz Bruker Avance-500 spectrometer (64 scans averaged per spectrum) with chemical shifts reported relative to residual $CHCl_3$.

Gel permeation chromatography (GPC). Polymer molecular weights and polydispersities were determined using an Agilent 1260 Infinity set-up comprising two Polymer Laboratories PL gel 5 μm Mixed-C columns, a refractive index detector and a Varian 290-LC pump injection module operating at 60 °C. The GPC eluent was HPLC-grade DMF containing 10 mM LiBr at a flow rate of 1.0 mL min^{-1}. Calibration was conducted using a series of eight near-monodisperse poly(methyl methacrylate) standards (M_n = 625 to 2,480,000 g mol^{-1}).

Dynamic Light Scattering (DLS) studies were conducted using a Malvern Zetasizer NanoZS instrument on 0.10% w/w aqueous dispersions at 25 °C in disposable cuvettes at a fixed scattering angle of 173°. Intensity-average hydrodynamic diameters were calculated *via* the Stokes-Einstein equation using a non-negative least-squares (NNLS) algorithm. All data were averaged over three consecutive runs. A mean sphere-equivalent hydrodynamic diameter of 176 nm (PDI = 0.30) was determined.

Transmission Electron Microscopy (TEM). The $PGMA_{56}$-$PHPMA_{155}$ diblock copolymer aqueous worm gel was diluted 100-fold at 20 °C to generate a 0.10% w/w free-flowing dispersion. Copper/palladium TEM grids were surface-coated in-house to yield a thin film of amorphous carbon. The grids were then plasma glow-discharged for 30 seconds to create a hydrophilic surface. 10 μL of the 0.1 % w/w dispersion was adsorbed onto the freshly glow-discharged grids for 60 s and then

blotted with filter paper to remove excess solution. To stain the aggregates, a 9 μL drop of 0.75% w/w uranyl formate solution was soaked on the sample-loaded grid for 20 s and then carefully blotted to remove excess stain. The grids were then dried using a vacuum hose. Imaging was performed at 80 kV using a FEI Tecnai Spirit microscope equipped with a Gatan 1kMS600CW CCD camera.

Rheology Studies. Storage (G') and loss (G'') moduli were determined between 25 °C and 4 °C for the $PGMA_{56}$-$PHPMA_{155}$ diblock copolymer worm gel using a TA Instruments AR-G2 rheometer. A cone-and-plate geometry (40 mm 2° aluminium cone) was used for these measurements, which were conducted at a fixed strain of 1.0 % and an angular frequency of 1.0 $rad.s^{-1}$

Differential Scanning Calorimetry (DSC) was undertaken on a Mettler Toledo DSC 1 Star. Sample (10 mg) of hydrogel suspension was cooled from 5 °C at 2 $°C.min^{-1}$ to -50 °C and then warmed back to 5 °C at 5 $°C.min^{-1}$.

Bright field and fluorescence microscopy were performed on an Olympus CKX41 with MoticamPro 205C and CoolLed pE-300-W light source, images analyzed using Motic Images Advanced 3.2. Cells used were screened, pathogen-free, defibrinated sheep red blood cells in Alsever's solution. All incubations of red blood cells were visualised at 1/10 dilution in PBS, with 2 μL solution on a glass slide with a glass cover slip.

Absorbance measurements were undertaken on a microplate reader (Synergy HT multi-mode microplate reader, BioTek UK).

Ice recrystallization inhibition (splat) assay. Ice recrystallization inhibition was measured using a modified splay assay.[1] A 10 μL sample of hydrogel dissolved in PBS buffer (pH 7.4) was dropped 1.40 m onto a chilled glass coverslip placed on a

piece of polished aluminum placed on dry ice. Upon hitting the chilled glass coverslip, a wafer with diameter of approximately 10 mm and thickness 10 μm was formed instantaneously. The glass coverslip was transferred onto the Linkam cryostage and held at −8 °C under N_2 for 30 minutes. Photographs were obtained using an Olympus CX 41 microscope with a UIS-2 20×/0.45/∞/0–2/FN22 lens and crossed polarizers (Olympus Ltd, Southend on sea, UK), equipped with a Canon DSLR 500D digital camera. Images were taken of the initial wafer (to ensure that a polycrystalline sample had been obtained) and again after 30 minutes. Image processing was conducted using Image J, which is freely available. In brief, ten of the largest ice crystals in the field of view were measured and the single largest length in any axis recorded. This was repeated for at least three wafers and the average (mean) value was calculated to find the largest grain dimension along any axis. The average of this value from three individual wafers was calculated to give the mean largest grain size (MLGS). This average value was then compared to that of a PBS buffer negative control providing a way of quantifying the amount of IRI activity.

Biological Assays

Cryopreservation of RBCs. Diblock copolymer worm hydrogel was resuspended in PBS buffer at 10 wt% at 3 °C inside a standard laboratory fridge. A 250 μL aliquot of freshly prepared ovine RBCs (packed cell volume between 32 and 52 %) was added to 250 μL of a cryosolution (containing hydrogel) or HES (350 $mg.mL^{-1}$), mannitol (30 $mg.mL^{-1}$) sodium chloride (6.5 $mg.mL^{-1}$)) and vortexed gently to ensure mixing. PVA was added at 1 $mg.mL^{-1}$ to the cryosolution in order to determine whether any beneficial effect could be seen. These were then rapidly frozen in triplicate by immersion into liquid nitrogen and stored within a liquid nitrogen container for 5 days.

Thawing was performed by placing in the fridge at 4 °C. Upon warming to room temperature regelation was observed.

Measurement of Red Blood Cell haemolysis and cell recovery. A 250 μL aliquot of red blood cell (RCB) / cryoprotectant solution was centrifuged in a 1.5 mL Eppendorf tube for 5 minutes at 6000 rpm in a temperature-controlled centrifuge at 3 °C. Then 10 μL of the supernatant was removed and added to 90 μL of PBS in a well of a 96-well plate. Absorbance was measured at 450 nm and compared to an unfrozen positive control containing 250 μL prepared RCBs and 250 μL PBS buffer. 100 % hemolysis samples were prepared by adding 250 μL RCBs to distilled water. Cell recovery was calculated by subtracting the attained haemolysis (%) from 100 (%) giving a figure for cell recovery (%).

Materials and Methods

Glycerol monomethacrylate (GMA; 99.8%) was donated by GEO Specialty Chemicals (Hythe, UK) and was used without further purification. 2-Hydroxypropyl methacrylate (HPMA; Alfa Aesar, UK), 2-cyano-2-propyl dithiobenzoate (CPDB; Sigma-Aldrich) and 4,4'-azobis(4-cyanopentanoic acid) (ACVA or V-501, 99%; Sigma-Aldrich, UK), poly(vinyl alcohol) (98 % hydrolyzed, Mw 13,000-23,000), Mannitol, hydroxyethyl starch (HES, 180 kDa), sodium chloride, (all Sigma-Aldrich, UK) were used as received. Ovine red blood cells in Alserver's solution were purchased from TCS Biosciences (UK). Phosphate-buffered saline (PBS) solution was prepared using pre-formulated tablets (Sigma-Aldrich) in 200 mL of Milli-Q water (>18.2 Ω mean resistivity) to give [NaCl] = 0.138 M, [KCl] = 0.0027 M, and pH 7.4.

Synthesis of $PGMA_{56}$ macro-CTA *via* RAFT solution polymerization

GMA (40.0 g, 250 mmol), CPDB (1.105 g, 5.0 mmol; target DP = 50), and ACVA (0.280 g, 1.00 mmol; PETTC/ACVA molar ratio = 5.0) were accurately weighed into a 250 mL round-bottomed flask. Anhydrous ethanol (previously purged with nitrogen for 1 h) was then added to produce a 40 % w/w solution, which was placed in an ice bath and purged under nitrogen for 45 min at 0 °C. The sealed flask was immersed in an oil bath set at 70 °C to initiate the RAFT polymerization of GMA and the polymerizing solution was stirred for 2 h at this temperature. The polymerization was then quenched at approximately 76% conversion by exposure to air, followed by cooling the reaction mixture to room temperature. Methanol (20 mL) was added to dilute the reaction solution, followed by precipitation into a ten-fold excess of dichloromethane in order to remove unreacted GMA monomer. The precipitate was isolated *via* filtration and washed with excess dichloromethane before being dissolved in methanol (60 mL). The crude polymer was precipitated for a second time by addition to excess dichloromethane and isolated *via* filtration. It was then dissolved in water and freeze-dried overnight to afford a pink solid. ^{1}H NMR studies indicated a mean degree of polymerization of 56 *via* end-group analysis while DMF GPC studies (refractive index detector; calibration *via* a series of near-monodisperse poly(methyl methacrylate) standards) indicated an M_n of 16,100 g mol^{-1} and an M_w/M_n of 1.11.

Synthesis of $PGMA_{56}$-$PHPMA_{155}$ diblock copolymer worm gel

The $PGMA_{56}$-$PHPMA_{155}$ diblock copolymer worm gel was prepared *via* polymerization-induced self-assembly (PISA) using the following RAFT aqueous dispersion polymerization formulation. $PGMA_{56}$ macro-CTA (0.583 g, 0.063 mmol),

HPMA monomer (1.40 g, 9.7 mmol) ACVA (5.9 mg, 0.021 mmol; $PGMA_{56}$ macro-CTA/ACVA molar ratio = 3.0) were added to a 25 ml round-bottomed flask, followed by the addition of water to produce a 10% w/w aqueous solution. This reaction solution was purged with nitrogen gas for 30 min at 20 °C before immersion into an oil bath set at 70 °C. The reaction mixture was stirred for 4 h to ensure essentially complete conversion of the HPMA monomer (> 99 % as judged by disappearance of the vinyl signals ay 6.1 ppm using ^{1}H NMR analysis) and was quenched by exposure to air, followed by cooling to ambient temperature. DMF GPC analysis indicated that the diblock copolymer possessed a relatively narrow polydispersity (M_w/M_n = 1.10) and a number-average molecular weight (M_n) of 43,300 g mol^{-1} (versus a series of near-monodisperse poly(methyl methacrylate) standards). The resulting worm dispersion was characterized by DLS, TEM and rheology without further purification.
